# Optimizing Graphene Ring Modulators: A Comparative Study of Straight, Bent, and Racetrack Geometries

**DOI:** 10.3390/nano15151158

**Published:** 2025-07-27

**Authors:** Pawan Kumar Dubey, Ashraful Islam Raju, Rasuole Lukose, Christian Wenger, Mindaugas Lukosius

**Affiliations:** 1 IHP-Leibniz Institut für Innovative Mikroelektronik, Im Technologiepark 25, 15236 Frankfurt (Oder), Germanylukose@ihp-microelectronics.com (R.L.);; 2 Semiconductor Materials, Brandenburg University of Technology Cottbus-Senftenberg, Platz der Deutschen Einheit 1, 03046 Cottbus, Germany

**Keywords:** graphene electro-absorption modulator, ring resonator, silicon nitride waveguide, finite-difference time-domain (FDTD) simulation, critical coupling

## Abstract

Graphene-based micro-ring modulators are promising candidates for next-generation optical interconnects, offering compact footprints, broadband operation, and CMOS compatibility. However, most demonstrations to date have relied on conventional straight bus coupling geometries, which limit design flexibility and require extremely small coupling gaps to reach critical coupling. This work presents a comprehensive comparative analysis of straight, bent, and racetrack bus geometries in graphene-on-silicon nitride (Si_3_N_4_) micro-ring modulators operating near 1.31 µm. Based on finite-difference time-domain simulation results, a proposed racetrack-based modulator structure demonstrates that extending the coupling region enables critical coupling at larger gaps—up to 300 nm—while preserving high modulation efficiency. With only 6–12% graphene coverage, this geometry achieves extinction ratios of up to 28 dB and supports electrical bandwidths approaching 90 GHz. Findings from this work highlight a new co-design framework for coupling geometry and graphene coverage, offering a pathway to high-speed and high-modulation-depth graphene photonic modulators suitable for scalable integration in next-generation photonic interconnects devices.

## 1. Introduction

The growing demand for high-speed, energy-efficient, and high-volume data transmission has driven research toward compact, scalable, and cost-effective optoelectronic devices. Advances in materials and fabrication technologies have enabled micro-scale waveguides, paving the way for chip-scale optical interconnects. Optical modulators, essential components in modern communication systems, were initially developed using silicon due to its availability and early adoption [1,2]. However, silicon’s weak electro-optic properties result in larger device footprints and higher energy consumption. This has motivated the exploration of alternative waveguide platforms such as silicon nitride (Si_3_N_4_), which offers ultra-low optical loss, a broad transparency window, and strong compatibility with CMOS fabrication processes. At the same time, researchers are increasingly investigating 2D material-based photonic structures—particularly graphene electro-absorption modulators—due to graphene’s broadband tunable absorption, high electrical conductivity, and strong nonlinear optical response [3,4,5,6,7,8]. Liu et al. first demonstrated a single-layer graphene straight modulator achieving 1 GHz speed by tuning the Fermi level electrically [9]. Straight modulator designs based on monolayer or bilayer graphene suffer from limited modulation depth and bandwidth due to weak light–graphene interaction [10,11,12,13,14,15,16]. To enhance this interaction, graphene has been integrated into ring resonators, which offer resonant enhancement and increased effective interaction length. Midrio et al. introduced a critically coupled graphene-based modulator, demonstrating tunable optical conductivity [17], followed by Qiu et al. and Phare et al., who reported electro-optic modulators using resonator loss modulation under critical coupling [18,19]. Graphene-based resonant structures have also been explored for compact tunable filters and passive photonic components, such as in mid-infrared planar plasmonic ring resonators [20]. However, most prior research on all-pass graphene ring modulators has employed a straight bus micro-ring configuration. While this represents a conceptually simple design, it can impose limitations on key performance parameters. In particular, such geometries often exhibit modest coupling efficiency (CE) and may require narrow coupling gaps, which in turn influence critical modulation characteristics such as bandwidth and extinction ratio. These constraints arise from inherent trade-offs in coupling geometry, where achieving strong interaction between the bus and the ring without incurring significant insertion loss remains a challenge. Therefore, while graphene-based ring modulators have been previously studied, the influence of bus–ring geometry on coupling conditions, modulation depth, and the effect of graphene length variation across different configurations is still insufficiently addressed. In particular, alternative bus geometries that support higher coupling efficiency—such as bent and racetrack designs—and their modulation behavior under varying graphene interaction lengths have received comparatively little attention, especially in the context of CMOS-compatible silicon nitride (Si_3_N_4_) photonic platforms. By combining graphene with a Si_3_N_4_ waveguide, this work presents a detailed comparative study of three resonator geometries—straight, bent, and racetrack—to identify design configurations that maximize modulation efficiency and robustness. This approach introduces new flexibility in the design of graphene-based modulators and enables performance optimization beyond the limitations of conventional straight bus configurations. A combination of finite-difference time-domain (FDTD) and eigenmode simulations is used to map the design space in terms of the coupling gap, graphene interaction length, and coverage. Our findings show that progressively lengthening the coupling region—from a straight bus to a bent section and ultimately to a racetrack configuration—shifts the critical coupling condition to larger gap values, while preserving high modulation depth and an overall improved figure of merit. Sensitivity analysis further reveals that covering only 6–12% of the circumference with graphene maximizes the extinction ratio while making the device tolerant to ±15% variations in graphene length, while also maintaining a large photon-lifetime-limited bandwidth. Implementing these insights, a 30 µm radius racetrack modulator is designed which achieves 28 dB extinction with a 10 V swing (corresponding to a modulation efficiency of 1.75 dB/V) and a projected 90 GHz bandwidth, while requiring graphene coverage on only ~12% of the ring circumference.

## 2. Methodology

**Figure 1:**
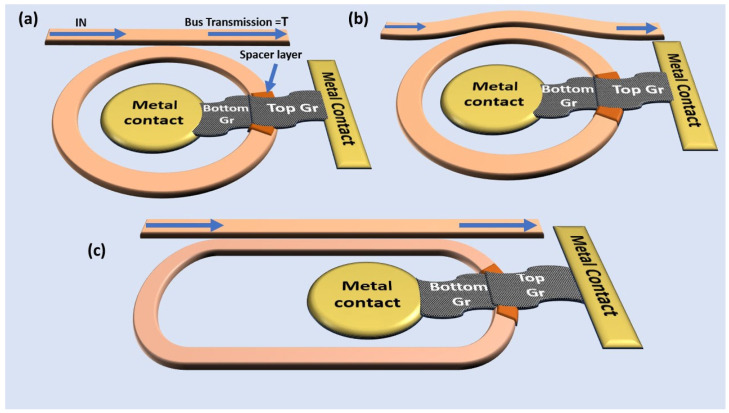
(**a**) Illustration of a bilayer graphene ring modulator with (**a**) single straight bus waveguide. (**b**) Ring modulator featuring a bent bus waveguide. (**c**) Racetrack graphene ring modulator.

Three modulator structures were investigated, namely the straight bus–ring modulator, the bent bus–ring modulator, and the racetrack modulator, as illustrated in Figure 1a–c. The core waveguide material was Si_3_N_4_, modeled using refractive index values corresponding to a 450 nm thick Si_3_N_4_ film. The parameters such as coupling efficiency, propagation loss, and graphene absorption were calculated for the transverse electric (TE) guided light mode at a wavelength of 1310 nm.

**Figure 2:**
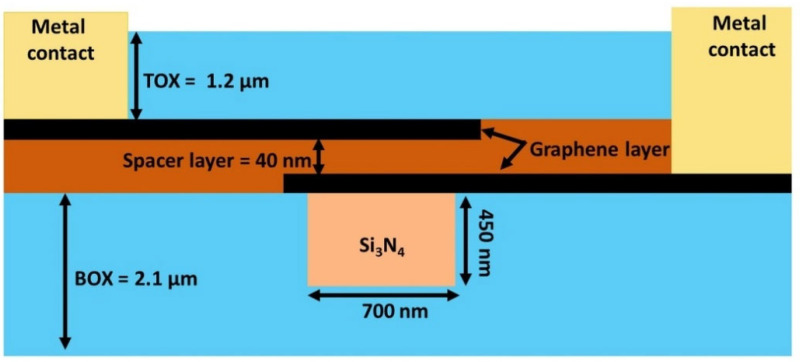
Schematic representation of the cross-section of a bilayer graphene modulator.

To ensure accuracy, perfectly matched layer (PML) boundary conditions were applied in the FDTD solver for coupling efficiency calculations and in the Mode solver for evaluating propagation loss. Furthermore, the graphene surface conductivity material model was utilized to accurately calculate the graphene’s absorption of the guided light mode. Figure 2 sketches the cross-sectional view of a bilayer graphene modulator applied to all the geometries depicted in Figure 1. Si_3_N_4_ has been used as the core waveguide material, with dimensions of 450 nm in height and 750 nm in width. The top oxide (TOX) and bottom oxide (BOX) act as the cladding layer for the waveguide core, with heights of 1.2 µm and 2.1 µm, respectively. The two-graphene layers are separated by a 40 nm dielectric layer, and a gold metal electrode was employed to apply the voltage to the individual graphene layer. Practical integration of high-quality graphene on silicon nitride has been demonstrated using advanced transfer and growth techniques such as dry transfer and direct chemical vapor deposition (CVD), as explained in recent studies [21,22], including controlled PECVD-based dielectric deposition after surface pre-treatment and annealing, which enables compatibility with standard fabrication flows [23,24].

The gate voltage V is applied to these metal contacts to adjust the absorption state of the graphene layer by modulating its chemical potential μ, according to the following relation:

(1) $$\mu \;\;=\hbar \;V_{F}\sqrt{\eta \pi \left|V-V_{dirac}\right|}\;$$

where ℏ is the reduced Plank constant, $V_{dirac}$ is the voltage offset caused by natural doping of the graphene layer, and $V_{F}=3\times 10^{8}\;m\cdot s^{-1}$ The shift in the chemical potential resulting from the applied voltages changes the optical conductivity $\sigma \left(\omega ,\mu ,\Gamma \right)$ of the graphene sheet expression, which is derived from the Kubo formalism [25]:

(2) $$\begin{array}{c} \left(\omega ,\mu ,\Gamma \right)=\frac{-ie^{2}}{\pi \hbar^{2}\left(\omega +i2\Gamma \right)}\int_{0}^{\infty }\xi \left(\frac{\partial f_{d}\left(\xi \right)}{\partial \xi }-\frac{\partial f_{d}\left(-\xi \right)}{\partial \xi }\right)d\xi \\ +\frac{\begin{array}{c} ie^{2}\left(\omega +i2\Gamma \right) \end{array}}{\pi \hbar^{2}}\int_{0}^{\infty }(\frac{f_{d}\left(-\xi \right)-f_{d}\left(\xi \right)}{{(\omega +i2\Gamma }^{2})-4{\left(\frac{\xi }{h}\right)}^{2}})d\xi \; \end{array}$$

where *ω* is the angular frequency, *Γ* is the scattering rate, *μ* is the chemical potential, *T* is the temperature, *e* is the electron charge, $\kappa_{B}$ is the Boltzmann constant, ξ is the energy level, and $f_{d}$ is the Fermi Dirac distribution function given by the following:

(3) $$f_{d}\equiv \frac{1}{\exp\left(\frac{\xi -\mu }{\kappa_{B}T}\right)+1}\;\;\;\;\;$$

The variation in the graphene’s optical conductivity, as per Equation (2), results in variation in the dielectric constant ε. Consequently, the waveguide’s effective refractive index *n* also changes, as indicated in Equation (4) below:

(4) $$n=\sqrt{\varepsilon }=\sqrt{1+\frac{\iota \sigma }{\omega \varepsilon_{0}d_{g}}}\;\;\;\;\;\;$$

**Figure 3:**
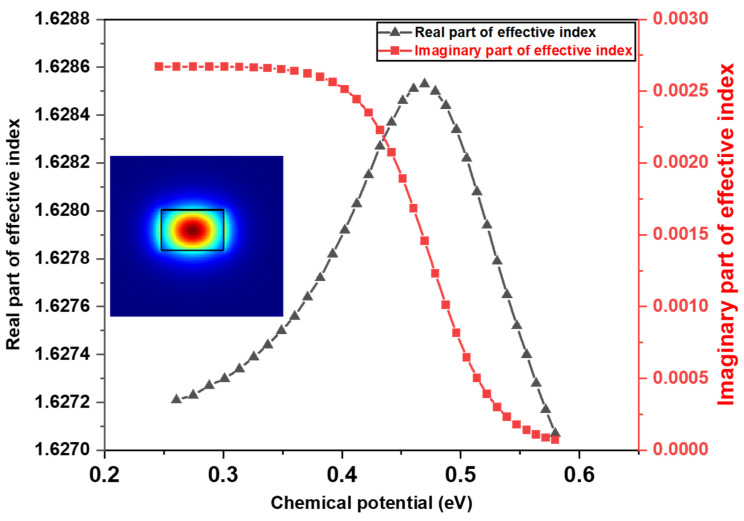
The variation in the real and imaginary part of the effective index with chemical potential for TE mode (inset).

Here, spacer layer thickness $d_{g}$ between the graphene sheets was chosen as 40 nm, which offers a balance between strong field overlap (for efficient modulation) and prevention of dielectric breakdown. Graphene was modeled as a 2D conductive sheet using the surface conductivity approach derived from the Kubo formalism. Specifically, we used Lumerical MODE’s eigenmode solver to compute the complex effective refractive index $n_{eff}$ of the graphene-integrated silicon nitride waveguide for different values of chemical potential μ (calculated from Ansys charge solver). The resulting $n_{eff}$ values were then used as input to emulate the electro-absorption modulation mechanism by varying μ as a function of applied gate voltage. The scattering rate Γ was fixed at 15 meV in all simulations, a value calculated based on experimentally derived parameters for CVD-grown graphene on silicon nitride, as reported in Wang et al. [26]. By using the Ansys charge solver, the graphene layer’s total charge and resulting chemical potential and optical conductivity σ for Equation (4) were calculated. These values are then utilized to compute the effective refractive index of the graphene-integrated silicon nitride waveguide as shown in Equation (3). In Figure 3, the variation in the real and imaginary components of the effective index for a graphene-on-silicon nitride waveguide system as a function of the chemical potential for TE mode (mode profile is shown in the inset) is plotted. These values were obtained using a mode solver’s finite-difference eigenmode (FDE) simulations. The extracted parameters were subsequently utilized to estimate the propagation loss in the waveguide due to the graphene’s absorption.

## 3. Results and Discussion

**Figure 4:**
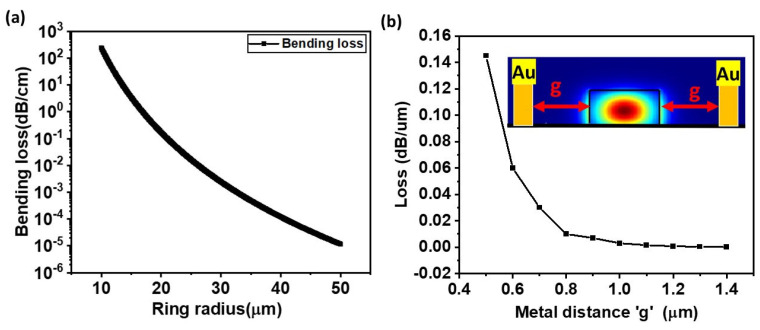
(**a**) Simulated radiation loss of the fundamental TE mode in a Si_3_N_4_ ring at 1310 nm. (**b**) Simulated excess attenuation per unit length introduced by gold electrodes. The inset picture shows the separation distance d and the TE mode propagation in the SIN core waveguide.

Using an FDTD eigen mode solver, the bending loss of the micro ring was first analyzed to design a resonator radius that provides an optimal balance between minimal radiation loss and a compact device footprint. Such a loss arises due to the tight curvature pushing the guided mode away from the waveguide’s high-index core; the resulting phase mismatch lets energy radiate into the surrounding cladding, so smaller radii leak light and degrade as it propagates in the waveguide core [27]. Simulations were carried out for the fundamental TE mode at λ = 1310 nm. The bend radius was swept from 10 µm to 50 µm while all other waveguide dimensions remained fixed. The resulting attenuation, plotted in Figure 4a, drops exponentially with the radius of the ring. Propagation losses remain below 0.2 dB/cm for a ring radius of 30 µm and become negligibly small beyond 40 µm. Based on this observation, a radius of r = 40 μm is selected for all subsequent designs, as it effectively suppresses radiation losses while preserving a suitable free spectral range for modulation and maintaining a compact device footprint. Secondly, metal-induced loss was evaluated by analyzing the overlap between the evanescent tail of the guided TE mode and the lossy metal electrodes [28]. The metal–waveguide separation ‘g’ (as illustrated in the inset of Figure 4b) serves as a critical design parameter, as it simultaneously influences the device’s electrical bandwidth and the optical loss induced by the metal. Reducing the gap lowers the overall resistance capacitance (RC) time constant and therefore extends the device’s electrical bandwidth; however, bringing the metal too close to the waveguide also increases parasitic absorption in the ring. An eigenmode solver was used to model two 360 nm thick gold (Au) metal contacts placed on either side of the waveguide (see Figure 4b, inset), with their lateral separation g swept from 0.5 µm to 1.4 µm. At g = 0.5 µm, the evanescent tail overlaps strongly with the metal, adding additional absorption of ≈0.15 dB/µm. The absorption decreases rapidly with the increase in distance ‘g’, and the value reaches 0.02 dB/µm at g = 0.7 µm, dropping below 0.01 dB/µm once the electrodes are ≥0.8 µm apart. Positioning the electrodes at least 0.8 µm from the core limits metal-induced attenuation to <0.01 dB/µm while maintaining a sufficiently smaller metal–graphene distance for high electrical bandwidth of the device.

**Figure 5:**
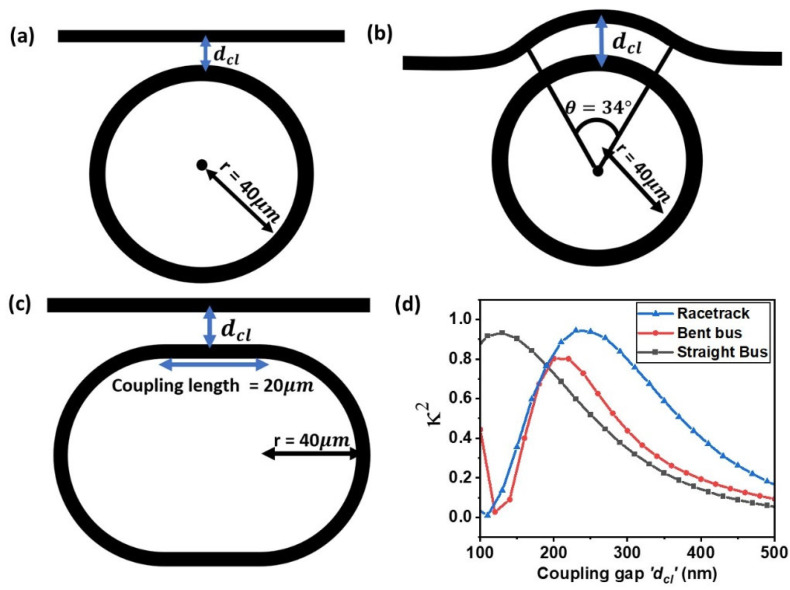
(**a**) Schematic of a straight bus waveguide coupled to a circular ring or radius r = 40 μm and coupling gap $d_{cl}$. (**b**) Bent bus design, where bus waveguide follows an arc of angle θ, enlarging the coupling region. (**c**) A racetrack structure design with 20 µm coupling length. (**d**) Comparison of coupling efficiency across different waveguide structures at a fixed ring radius of 40 µm and a wavelength of 1310 nm.

Moreover, the bus–ring power coupling efficiency $\kappa^{2}$ was subsequently evaluated, where $\kappa^{2}=1$ corresponds to complete power transfer between the waveguide and the ring (output bus transmission T = 0). This parameter is tunable via the gap between the ring and the bus waveguide. A FDTD simulation for three evanescent-coupled resonator layouts—a straight bus waveguide adjacent to a circular ring (Figure 5a), a bent bus waveguide coupled to the ring (Figure 5b), and a racetrack resonator with a straight coupling section (Figure 5c)—were conducted. Adapting the standard formulation of coupled mode theory for two weakly coupled, phase-matched optical waveguides [29,30], the slowly varying field envelopes in the bus, a(z), and in the ring, b(z), can be written as follows:

(5) $$\frac{da}{dz}=-jk_{c}b\frac{db}{dz}=-jk_{c}a-j\frac{\Delta \beta }{2}b\;\;\;$$

where a and b are the normalized complex amplitude of the guided mode (travelling along z) in the bus and ring, respectively, and β is the propagation constant. The per length coupling constant falls off almost exponentially with the bus–ring gap d as $\left(d\right)=k_{0}exp\left(-d/d_{ev}\right)$ with $d_{ev}$ is evanescent-field decay length into the cladding. Integrating Equation (5) over the physical interaction length L gives the following complex field-transfer coefficient:

(6) $$\left(L\right)=\;\frac{\kappa_{C}}{\sqrt{\kappa_{c}^{2}+{\left(\Delta \beta /2\right)}^{2}}}sin\left[\sqrt{\kappa_{c}^{2}+{\left(\Delta \beta /2\right)}^{2}}\;L\right]$$

and the power-coupling efficiency is given by ${\left|\kappa \left(L\right)\right|}^{2}$. In the common phase-matched Δβ = 0, the coupling efficiency is given by the following:

(7) $${\left|\kappa \left(L\right)\right|}^{2}=\;{sin}^{2}\left[k_{c}\left(g\right)L\right]$$

Here, the only geometry-dependent parameter is the interaction length or the coupling length L. For the straight bus coupled to the ring, the coupling length $L_{s}$ is shorter in comparison to the bent bus coupling length $L_{b}$ = rθ and the racetrack coupling length $L_{RT}$. Although the bent bus coupler offers a nominally longer interaction length ($L_{b}$ = R × θ = 26 μm) for a 40 µm radius ring and an arc angle of 34° (Figure 5b), its power-coupling efficiency is nonetheless lower than that of the racetrack, whose straight coupling section is only 20 μm (Figure 5d). This can be explained by the coupled-mode expression (Equation (6)), showing that coupling efficiency depends not on length alone but on the combined product $\kappa_{C}L$ and L and the phase-mismatch penalty ${\left(∆\beta /2\right)}^{2}$. In the bent bus, the curved waveguide’s mode maximum is located further from the ring core, so the per-length coupling coefficient $\kappa_{C}$ is intrinsically smaller than in the parallel, straight-guide section of the racetrack. Curvature also introduces a non-zero propagation-constant difference Δβ = $\beta_{bus}-\beta_{ring}$, further suppressing the overall coupling efficiency relative to the racetrack coupling design. For each geometry shown in Figure 5, the bus–ring coupling gap separation $d_{cl}$ was swept from 100 nm to 500 nm, with the ring radius fixed at *r* = 40 µm. In the 2D finite-difference time-domain (FDTD) simulation, a power monitor is placed inside the micro-ring resonator. A broadband Gaussian pulse centered at a wavelength of 1310 nm with TE polarization is injected, and the resulting spectral response of the light confined within the ring is recorded. Figure 5d compares the coupling efficiencies for a 40 µm ring radius across all resonator geometries, depicting how each design—straight bus, bent bus, and racetrack—performs at the same ring radius. Overall, the results show that the straight and bent bus waveguide configurations achieve their highest coupling efficiency at moderate gaps (roughly 100–200 nm), whereas the racetrack design maintains more significant coupling efficiency at larger gaps (250–300 nm). Consequently, while smaller gaps are necessary to sustain strong coupling in the straight and bent bus geometries, a racetrack-based modulator design can still achieve high efficiency at gaps of 250 nm and beyond. This understanding is crucial, as coupling efficiency directly determines the resonator’s transmission characteristics, a key parameter for the operation of a ring modulator.

**Figure 6:**
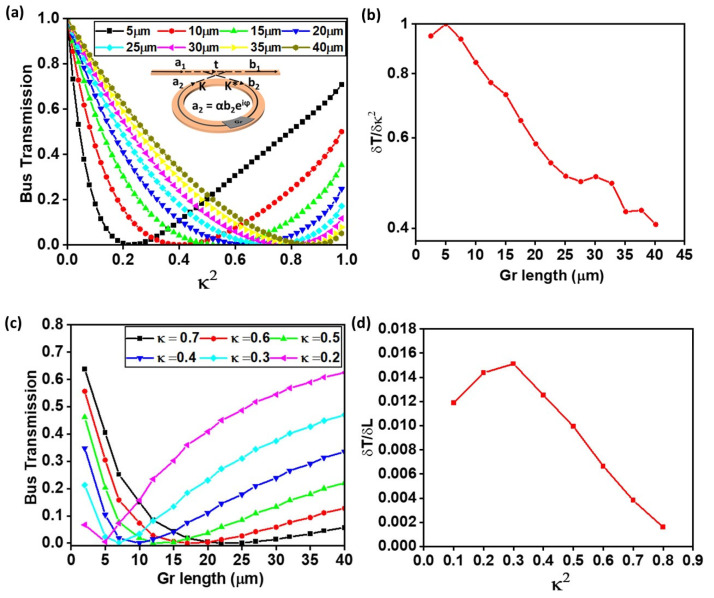
(**a**) Simulated bus transmission T v.s. $\kappa^{2}$ for a 40 µm radius ring resonator with varying graphene lengths from 5 µm to 40 µm. (**b**) Coupling sensitivity δT/δ$\kappa^{2}$ extracted from the slope of the curves in (**a**), showing the highest sensitivity for short graphene lengths. (**c**) Bus transmission T versus graphene length for various fixed coupling coefficients $\kappa^{2}$, illustrating that the length of graphene required to reach critical coupling decreases with increasing coupling strength. (**d**) Graphene length sensitivity δT/δ$L_{gr}$ as a function of coupling efficiency $\kappa^{2}$, highlighting that the transmission is most sensitive to changes in graphene length near the critical coupling regime.

The working principle of the graphene-integrated ring modulator is described using all-pass ring resonator formalism [31,32], which relates the input complex field amplitude $a_{1}$ and output complex field amplitude $b_{1}$ as shown in the inset of Figure 6a. Here, α is the round-trip loss coefficient of the ring, and t is the self-coupling coefficient of the ring–bus system. The former is fixed by the bus–ring coupling gap and length $d_{cl}$, whereas the latter is tuned electrically by changing the graphene absorption with gate voltage or tuning the graphene length itself. Assuming lossless coupling such that |${\left|t\right|}^{2}+{\left|\kappa \right|}^{2}=1$, where κ is the cross-coupling coefficient, the normalized bus output transmission T at resonance is given by the following:

(8) $$T={\left(\frac{b_{1}}{a_{1}}\right)}^{2}=\frac{\left(\alpha -\left|t^{2}\right|\right)}{\left(1-\alpha \left|t^{2}\right|\right)}\;\;\;\;\;\;$$

From Equation (8), it is evident that bus output transmission T is governed solely by the interplay between α and t.

(9) $$\alpha \equiv \alpha_{crit}=t\;$$

When the above condition is satisfied, the numerator in Equation (8) vanishes and total destructive interference leads to zero output bus transmission T, i.e., the critically coupled condition. On the other hand, when α→1, representing a nearly lossless ring (e.g., when graphene becomes highly transparent), the denominator dominates, and the bus transmission approaches unity.

This dynamic range in T, tunable via the gate-controlled modulation of α, enables effective intensity modulation. Therefore, by co-designing the coupling parameters (i.e., ring radius, coupling gap, and coupling length) and the graphene bias range, one can ensure the system reaches the critical coupling condition required to achieve high modulation depth in graphene ring modulators. Following this ring resonator theory, a graphene ring modulator with a straight bus was modeled in the simulation. The purpose is to study the output bus transmission with different graphene length sections on the ring circumference (inset of Figure 6a). In the simulations, only the magnitude of the coupling coefficient ($\kappa^{2}$) is used as an input parameter, based on frequency-dependent mode overlap characteristics imported from FDTD eigenmode solvers. For this reason, the results in Figure 6a were plotted only for the straight bus–ring configuration, as it sufficiently captures the dependence of extinction behavior on coupling gap parameter *κ*^2^ and the graphene length via the effective loss parameter α, without loss of generality across the other structures [33]. At first the graphene layer was gate-tuned to a chemical potential of 0 eV, placing it in a high conductivity state. This condition effectively rendered the graphene to an absorptive state, with a non-zero imaginary component of the effective index (as shown in Figure 3). Figure 6a presents the simulated bus transmission *T* at different coupling efficiency *κ*^2^ for various graphene lengths integrated on a ring with a 40 µm radius coupled to a straight bus waveguide. The transmission exhibits the minimum value for a specific value of *κ*^2^, which satisfies Equation (6), indicating the onset of critical coupling for each graphene length. Importantly, the critical coupling condition not only minimizes transmission, which is important for a high extinction ratio switching device, but also corresponds to the point where the modulation sensitivity, i.e., the derivative (∂T/∂a · ∂a/∂V), is maximized [19], offering the steepest response to changes in graphene properties. This makes it an ideal reference point to quantify the modulation effect as a function of physical design variables such as coupling gap or graphene length. Moreover, it is well understood that critical coupling does not always represent the optimal operating point for signal integrity in high-speed data transmission systems [33]. This regime is nonetheless selected for its utility in establishing a well-defined reference point for assessing intrinsic modulation characteristics. Analyzing near the critical coupling point enables a clear assessment of how the coupling efficiency and graphene-induced absorption influence key modulator figures of merit, such as the extinction ratio and insertion loss. As shown (Figure 6a), with the increase in graphene, the coupling efficiency required to achieve critical coupling also increases, shifting the transmission minimum toward a higher $\kappa^{2}$ value. This reflects that stronger absorption (i.e., lower α) necessitates stronger coupling to satisfy the critical condition. To quantify how sensitively the output transmission responds to variations in coupling conditions, the derivative *δT*/*δ*$\kappa^{2}$ was extracted and plotted as a function of graphene length (Figure 6b). The monotonic decrease in sensitivity *δT*/*δ*$\kappa^{2}$ with increasing graphene length indicates that modulators incorporating longer absorptive sections exhibit reduced dependence on precise coupling efficiency required for critical coupling. In other words, as the graphene interaction length increases, the output transmission becomes less sensitive to deviations from the ideal critical coupling condition, even under slight variations in the bus–ring coupling geometry that affect the parameter $\kappa^{2}$.

**Figure 7:**
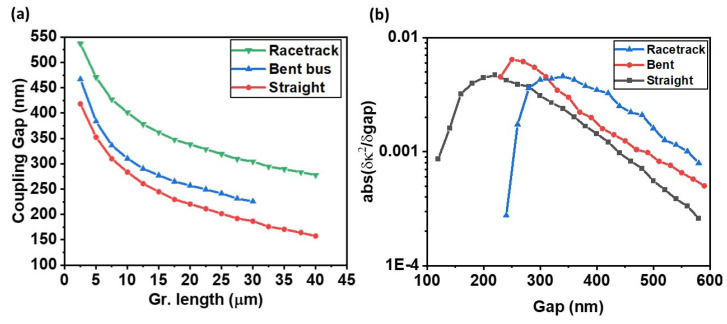
(**a**) Required coupling gap to achieve critical coupling as a function of graphene length for three bus–ring geometries: straight, bent, and racetrack. (**b**) Sensitivity of coupling efficiency with respect to coupling gap, expressed as (∂κ2/∂gap) for each geometry.

Figure 6c presents the variation in bus transmission as a function of graphene length for fixed coupling coefficients ranging from $\kappa^{2}=0.2\;to\;0.7$ to 0.7. As expected from the ring resonator theory, increasing the graphene length introduces greater optical absorption, resulting in a lower round-trip loss α. To satisfy the critical coupling condition α = t, the self-coupling coefficient t must also decrease accordingly. This implies that a higher coupling efficiency ($k^{2}=1-t^{2}$) is required to maintain critical coupling as the graphene length increases. Figure 6d quantifies the sensitivity of transmission to graphene length by plotting *δT*/*δ*L. The sensitivity curve peaks near the moderate coupling coefficient value and decreases significantly for the higher coupling value. This finding is particularly relevant for electro-absorption modulators, where the effective graphene length—or equivalently, its absorptive strength modulated via gate voltage—is a key tuning parameter. As shown in Figure 6d, the transmission sensitivity to graphene length δT/δ$L_{gr}$ reaches a maximum near the critical coupling regime. This implies that even slight deviations in graphene absorption length from the designed value can significantly alter the transmission characteristics, effectively shifting the critical point. While this high sensitivity is beneficial for achieving strong modulation contrast, it also reflects a narrower design margin, making the device performance more susceptible to variations—even slight changes in graphene length. Together, Figure 6c,d provide a comprehensive design space that captures the trade-offs between extinction ratio, modulation sensitivity, and structural robustness in graphene-integrated micro-ring modulators. Extending this analysis to find out the impact of coupler geometry, Figure 7a compares the required coupling gap to reach critical coupling for three different bus–ring configurations—straight, bent, and racetrack—as a function of graphene length.

The results reveal that for any given graphene length, the racetrack configuration consistently requires a larger coupling gap to achieve the same $k^{2}$ value, followed by the bent bus, while the straight bus yields the smallest required gap. This behavior is attributed to the mode overlap efficiency of each geometry: racetrack couplers, due to their extended interaction length and geometry-induced mode mismatch, require larger physical gaps to reach a specific coupling strength as illustrated in Figure 5d. Complementary to this, Figure 7b shows the sensitivity of the coupling efficiency (∂$k^{2}$/∂gap) for all three configurations. For all structures, a peak in sensitivity is observed at intermediate gap values, with racetrack designs showing a delayed peak due to their broader coupling region. This indicates that racetrack-coupled modulators can maintain high coupling efficiency even at increased gap values, offering enhanced design flexibility. As a result, such designs are able to maintain operation near the critical coupling point even in the presence of minor variations in the coupling gap, thereby improving overall device robustness without sacrificing modulation performance. The results presented here reinforce that each structure imposes a unique coupling profile, which in turn affects the required graphene length and achievable modulation contrast. Therefore, design optimization must be co-dependent on both the coupler layout and absorption characteristics, ensuring that the structure operation near the desired critical point has sufficient tolerance and efficiency. To comprehensively assess the modulation behavior at a fixed coupling gap of 300 nm across different bus configurations, transmission spectra were simulated for straight, bent, and racetrack-coupled micro-ring modulators, with each design tuned to operate near its critical coupling point. For each configuration, a range of gate-tunable graphene chemical potentials μ was applied—from 0 to 0.57 eV—to modulate absorption and hence the effective loss *α*. The performance was benchmarked using key figures of merit: modulation depth (MD), extinction ratio (ER), insertion loss (IL), resonance wavelength shifting between graphene on and off Δλ, full width at half maximum (FWHM), and the photon lifetime limited 3 dB bandwidth (BW), which is defined as follows:

(10) $$BW\;=\;\frac{1}{2\pi \tau_{ph}}\approx \frac{c}{\lambda Q}$$

where c is the light speed, $\lambda_{res}$ is the resonance wavelength, and Q is the loaded quality factor of the graphene ring modulators. Using these values, the overall figure of merit (FOM) is defined as follows:

(11) $$FOM=\;\frac{Modulation\;depth\;\left(MD\right)\times Bandwidth\;(BW)}{V_{PP}\times average\;Insertion\;loss\;({IL}_{avg})}\;\;\;\;\;$$

**Figure 8:**
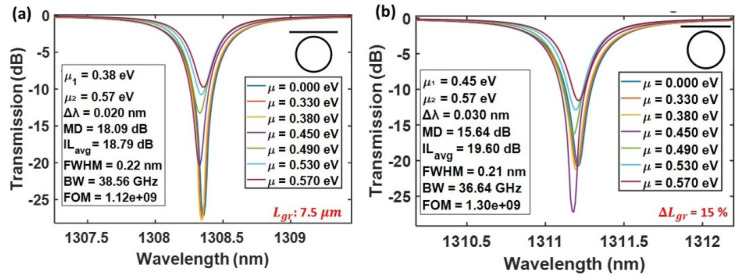

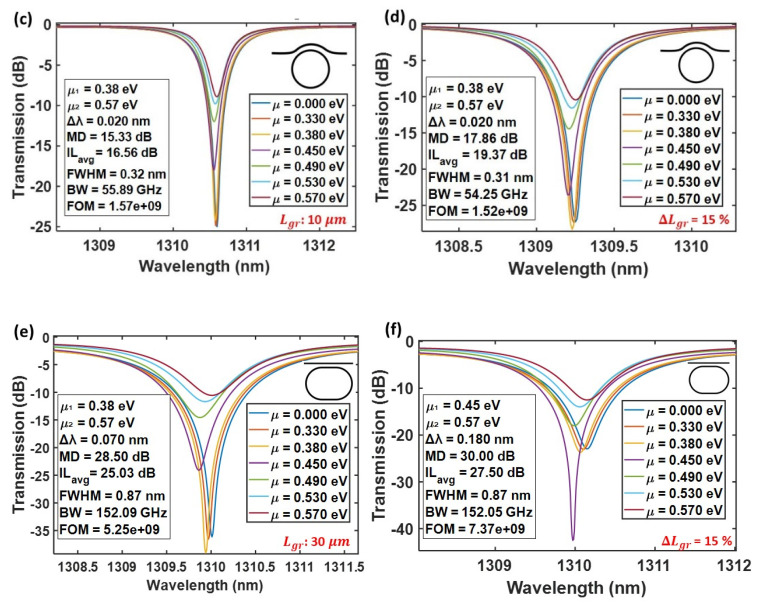
The transmission spectra of graphene-on-Si_3_N_4_ micro-ring modulators for three different bus geometries—straight, bent, and racetrack—each evaluated at the central graphene length required to achieve critical coupling and at a length 15% greater than this value. Graphs (**a**) and (**b**) correspond to the straight bus configuration with $L_{gr}$ = 7.5 µm and $L_{gr}$ = + 15%, respectively; (**c**,**d**) depict the bent bus design with $L_{gr}$ = 10 µm and its 15% extension, while (**e**,**f**) represent the racetrack geometry with $L_{gr}$ = 30 µm and the corresponding +15% increase. For each case, the effect of tuning the graphene chemical potential (**μ**) is illustrated, and the key performance metrics—extinction ratio, bandwidth, insertion loss, and figure of merit—are extracted.

**Figure 9:**
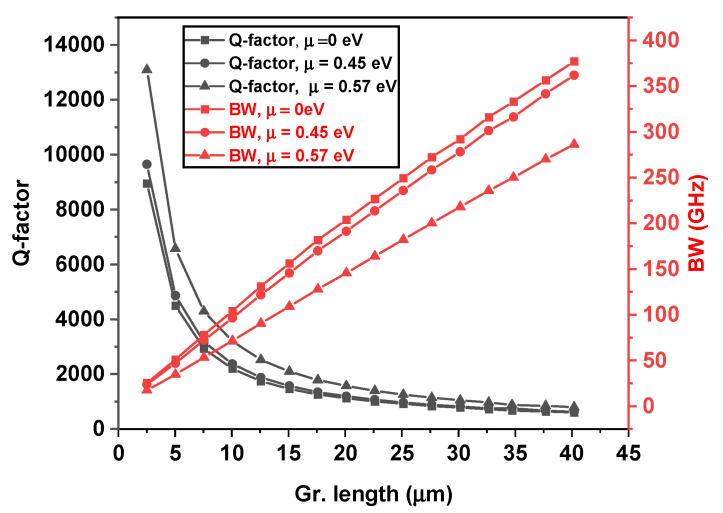
Loaded quality factor (black curve, left y-axis) and corresponding bandwidth (red curve, right y-axis) plotted as functions of graphene length for a ring radius of 40 µm.

In the straight bus configuration (Figure 8a,b), the graphene length was set to 7.5 µm for optimal critical coupling. Sweeping μ resulted in a maximum modulation depth of 18.09 dB and a narrow FWHM of 0.22 nm, with a corresponding BW of 38.66 GHz. The resonance wavelength shifted by ~0.020 nm with gating, and the average insertion loss (${IL}_{avg}$) between the ON and OFF states was ~19 dB. A 15% increase in graphene length caused a red shift in resonance (~2.85 nm), and slightly reduced ER, though the bandwidth remained comparable. In the bent bus configuration (Figure 8c,d), a longer graphene length of 10 µm was needed due to lower the coupling efficiency. The transmission spectrum showed broader FWHM (0.32 nm), larger BW (~65.89 GHz), and MD of 16.53 dB, with ${IL}_{avg}$~19.7 dB. Upon a 15% length increase, a wavelength red shift of ~0.25 nm occurred, and modulation performance remained largely stable. The FOM stayed consistent around 1.5 × 109, slightly higher than the straight bus design. In the racetrack configuration (Figure 8e,f), a graphene length of 30 µm achieved deep modulation with MD ~28.50 dB, ${IL}_{avg}$ ~26 dB, and a wide BW of 162.09 GHz. The variation in photon life limited bandwidth coming from the quality factor of the resonator and at different chemical potentials of the graphene is plotted in Figure 9. Thus, enhanced bandwidth is primarily attributed to the reduced quality factor of the resonator, owing to larger graphene section as shown in Figure 9.

Notably, introducing a 15% increase in graphene length in the racetrack structure (Figure 8f) did not degrade the modulation depth or significantly shift the resonance wavelength, demonstrating robustness in performance, which was observed. However, to maintain optimal contrast for slightly deviated graphene length, a different chemical potential μ had to be selected for the ON state. This observation suggests that minor adjustments in gate voltage can compensate for physical variations in the graphene length, effectively preserving key modulation parameters such as MD, bandwidth, and resonance alignment. Thus, racetrack geometries not only enable high-performance modulation but also present the potential for voltage-level compensation strategies to mitigate performance degradation arising from variations in physical parameters such as coupling gap and graphene length. Moreover, the FOM is overall larger in racetrack than the straight and bent bus designs and further improves from 5.25 × 109 to 7.37 × 109 when the graphene length is changed, reflecting unchanged performance with even a 15 percent deviation in graphene length.

Since no direct study on bus geometry’s dependence on critical coupling gaps and modulation depth has been reported to date, the performance of the proposed racetrack modulator can still be compared with previously published works. For instance, Lee et al. [34] demonstrated a graphene–silicon nitride electro-absorption ring modulator with a 180 nm coupling gap where a modulation depth of 7 dB at 9 V was achieved. By contrast, the racetrack design proposed here, which features a much wider 300 nm coupling gap, achieves an approximately four times higher modulation depth (28 dB over 10 V). Furthermore, experimental data from Phare et al. [19] indicate a 15 dB modulation depth at a 10 V drive voltage. In contrast, the device presented in this work demonstrates 30 dB modulation depth under the same drive conditions, effectively doubling Phare et al.’s result. In another study by Faneca et al. [35], the modulator reached a modulation depth of 16.5 dB at a 10 V swing, with the values two times lower than the racetrack modulator.

As voltage-dependent performance has been evaluated, the following key parameter has to be considered: the bandwidth of the modulator.

**Figure 10:**
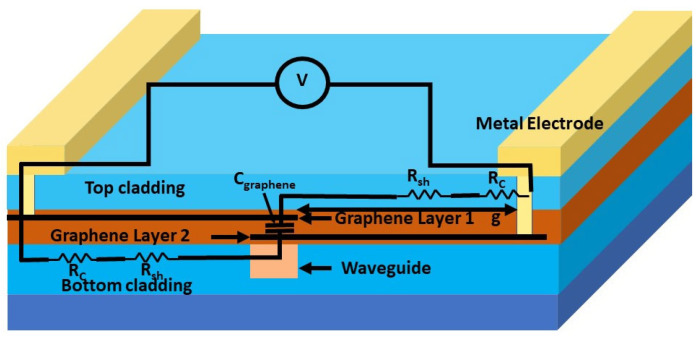
The cross-section views of the device with the equivalent RC circuit superimposed, illustrating the electrical component and their arrangement within the structure.

Figure 10 shows the cross-sectional view of the device overlaid with the various resistances and the capacitance used to estimate the bandwidth of the device. To fabricate the via contact to graphene, standard pilot-line-compatible processes involve etching vias through the dielectric using controlled wet or dry methods (e.g., hot phosphoric acid or low-power plasma), followed by metal deposition (e.g., Ni/TiN/W) to form low-resistance edge or surface contacts, as demonstrated in integrated graphene devices [36,37]. The RC time-limited 3 dB bandwidth of such configuration is obtained as follows:

(12) $$f_{3dB}=\;\frac{1}{2\pi RC}=\;\frac{1}{2\pi \left(2\frac{R_{C}}{l}+2R_{sh}\frac{g}{l}\right)\epsilon_{0}\varepsilon_{r}\frac{wl}{d}}$$

where $R_{C}$ is the contact resistance, $R_{sh}$ is the sheet resistance of the graphene, g = 0.8 μm is the distance between the metal electrode and the graphene capacitor, w is the graphene capacitor, d is the spacer layer thickness, and l = 30 μm is the graphene length. Nevertheless, it is clear from Equation (12) that the contribution of l coming from the sheet resistance and the graphene capacitance cancels out. Therefore, Equation (12) is reduced to the following:

(13) $$f_{3dB}=\;\frac{1}{2\pi \left(2R_{C}+2R_{sh}g\right)\varepsilon_{0}\varepsilon_{r}\frac{w}{d}}$$

**Figure 11:**
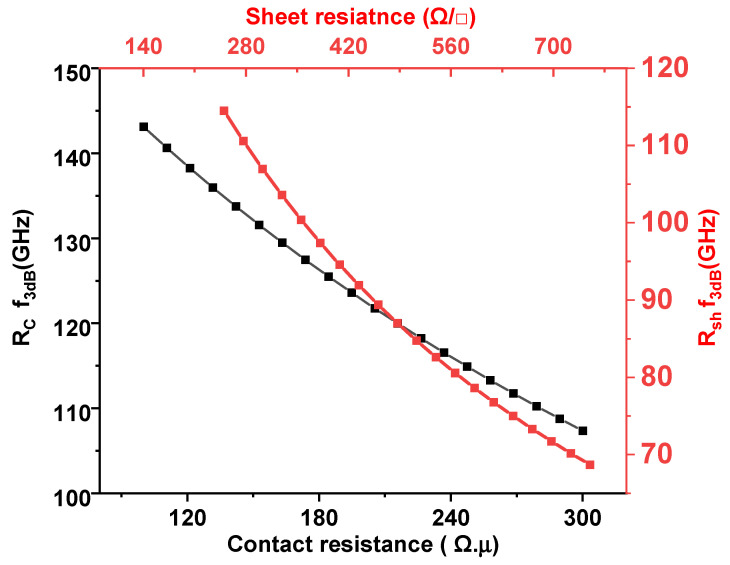
Plot of the 3 dB bandwidth versus contact resistance (black curve) with a fixed sheet resistance of 500 Ω/□ and 3 dB bandwidth vs. graphene sheet resistance (red curve) with a fixed value of the contact resistance of 200 Ω⋅μm.

Equation (13) is plotted in Figure 11 as a function of contact resistance. With the parameters values Rc=200 Ω⋅μm and $R_{sh}=500\;\Omega /□$ an RC-limited bandwidth of 91 GHz can be achieved. The bandwidth of the measured device remains below the photon-limited bandwidth of over 200 GHz for 30 µm long graphene (Figure 9), indicating that the RC bandwidth currently imposes the main limitation. Nonetheless, there is potential for further improvement in the overall RC modulation bandwidth by enhancing graphene quality and reducing metal contact resistance. Table 1 summarizes a comprehensive comparison of key performance metrics for dual-layer graphene ring modulators reported in the literature. To the best of our knowledge, no prior work has demonstrated a dual-layer graphene-based modulator with a bent bus coupling configuration operating on the principle of electro-absorption modulation.

Notably, the proposed racetrack modulator in this work achieves 90 GHz of overall modulation bandwidth—1.5 times the 60 GHz (without 50 Ω load resistor) demonstrated by Phare et al. [19] and four times higher than the 14.7 GHz reported by Lee et al. [34], both of which are experimental studies. This represents a significant improvement, even when compared to theoretical simulations: the racetrack modulator designed here surpasses the 42.6 GHz bandwidth of the straight bus modulator modeled by Neves et al. [38] by 18 GHz. Furthermore, while Neves et al. employed a 25% graphene coverage and a 205 nm coupling gap, the racetrack graphene modulator presented here achieves 28 dB of modulation depth with 12% graphene coverage and a wider 300 nm coupling gap yet still delivers a theoretical bandwidth of 90 GHz. This choice of 300 nm gap was intentionally made to ensure a consistent baseline for performance comparison with the straight and bent bus modulator designs, all evaluated under similar coupling sensitivity conditions. Notably, even at this relatively large gap, the racetrack configuration maintains a high coupling efficiency of approximately 0.78—substantially higher than what is typically achievable with a bent or straight bus configuration at the same gap. Moreover, due to the extended interaction length inherent in the racetrack geometry, the coupling efficiency remains sufficiently strong even at gaps as large as 400 nm, offering significant flexibility in fabrication without compromising performance. Therefore, even with the reduced coverage/area of the graphene, the device can still achieve modulation depths as high as 18 dB, highlighting the strength of the racetrack-based design and its potential for further optimization in high-speed and high-modulation-efficiency photonic devices.

## 4. Conclusions

This study provides key insights into the design of Si_3_N_4_ micro-ring modulators operating at the 1.31 µm wavelength by systematically comparing three resonator geometries—straight, bent, and racetrack. Using finite-difference time-domain (FDTD) simulations for each geometry, the influence of graphene length and coverage on resonance shaping and extinction ratio is quantified. At a fixed 300 nm gap and near-critical coupling, the racetrack design only requires 6–12 % graphene coverage to achieve an extinction ratio of ~28 dB (1.75 dB/V modulation efficiency) and a projected electrical bandwidth approaching 90 GHz. Furthermore, the racetrack modulator maintains stable modulation performance even under a 15% variation in graphene length, whereas the straight and bent configurations exhibit noticeable degradation in device response. These results establish a novel co-design framework linking coupling geometry, gap size, and graphene interaction length and identify the racetrack architecture as a highly promising approach for compact, high-extinction, and high-speed graphene modulators suitable for integrated photonic platforms. To our knowledge, this is the first comprehensive comparison of coupling geometries in graphene-on-Si_3_N_4_, offering new design strategies for achieving high extinction ratios and bandwidth with relaxed fabrication constraints related to coupling gap width and graphene length variations.

## Figures and Tables

**Table 1:** Comparison of performance metrics for graphene-based optical modulators reported in various journals.

Study	Platform	Geometry	Coupling Gap (nm)	Graphene Coverage/Length	Modulation Depth (dB)	3 dB Bandwidth (GHz)
Phare et al., Nat. Photonics, 2015 [19]	Si	Straight bus–ring	~180	16%/30 μm	15	60
Lee et al., Nanophotonics, 2021 [34]	Si_3_N_4_	Straight bus–ring	180	5 μm	7	14.7
Faneca et al., Opt. Express, 2019 [35]	Si-rich Si_3_N_4_	Straight bus–ring	250	11μm	16.5	15.6
Neves et al., Opt Quant Electron; 2019 [38]	Si	Straight bus–ring	-	-	<15	~40
Yang et al.. IEEE ICOCN, 2019 [39]	Si	Straight bus–ring	200–250	-	15.5	~16
Luan et al. Optica CLEO, 2022 [40]	Si	Racetrack	130	~25%	27.2	40
Neves et al., Opt. Continuum, 2022 [41]	Si_3_N_4_	Straight bus–ring	205	25%	-	42.6
This work	Si_3_N_4_	Bent bus–ring	340	4%/10 μm	15.33	50
This work	Si_3_N_4_	Racetrack	300	12%/30μm	28.5	90

## Data Availability

Data underlying the results presented in this paper are not publicly available at this time but may be obtained from the authors upon reasonable request.
